# Data for transcriptome and proteome analysis of *Eucalyptus* infected with *Calonectria pseudoreteaudii*

**DOI:** 10.1016/j.dib.2014.12.008

**Published:** 2015-01-09

**Authors:** Quanzhu Chen, Wenshuo Guo, Lizhen Feng, Xiaozhen Ye, Wanfeng Xie, Xiuping Huang, Jinyan Liu

**Affiliations:** a Sanming City Forest Disease and Pest Control and Quarantine Station, Sanming, Fujian 365000, China; b Forestry College, Fujian Agriculture and Forestry University, Fuzhou 350002, China; c Institute of Forestry Protection, Fujian Agriculture and Forestry University, Fuzhou 350002, China

## Abstract

*Cylindrocladium* leaf blight is one of the most important diseases in *Eucalyptus* plantations. We investigated the proteome and transcriptome of *Eucalyptus* infected or not infected with *Calonectria pseudoreteaudii*. Here we provide the information about the processing of raw data obtained by RNA-seq and iTRAQ technologies. The data are related to [1].

**Table:** Specifications table

Subject area	*Biology*
More specific subject area	*Forest pathology*
Type of data	*Table, figure*
How data was acquired	*RNA-seq:RNA isolation, Library construction and sequencing (Illumina), mapped to the sequenced genome of Eucalyptus grandis by SOAPaligner/soap2; iTRAQ:iTRAQ labeling (Applied Biosystems), SCX chromatography (Shimadzu), Q-Exactive MS (Thermo Fisher Scientific) and LC-20 AD nano HPLC (Shimadzu)*, *MASCOT search (Matrix Science, version 2.3.02) against E.grandis sequence database (JGI version 0.9, http:/phytozome.net/eucalyptus.php, 46,315 sequences)*
Data format	*Raw and analyzed*
Experimental factors	*Seedlings of Eucalyptus cultivated in the nursery were inoculated with Calonectria pseudoreteaudii and mock-inoculated with sterile water.*
Experimental features	*Total RNA sequencing (RNA-seq) and Isobaric tags for relative and absolute quantification (iTRAQ)*
Data source location	*Fuzhou, China*
Data accessibility	*The data is available with this article and is related to*[1]

**Value of the data**

• The data can be referenced by other scientists investigating the response mechanism of *Eucalyptus* to other stresses.
• The data can provide comprehensive analysis of proteome and transcriptome *of Eucalyptus* to *C. pseudoreteaudii.*

## 1 Data, experimental design, materials and methods

### 1.1 Plant material and experimental design

**Fig. 1:**
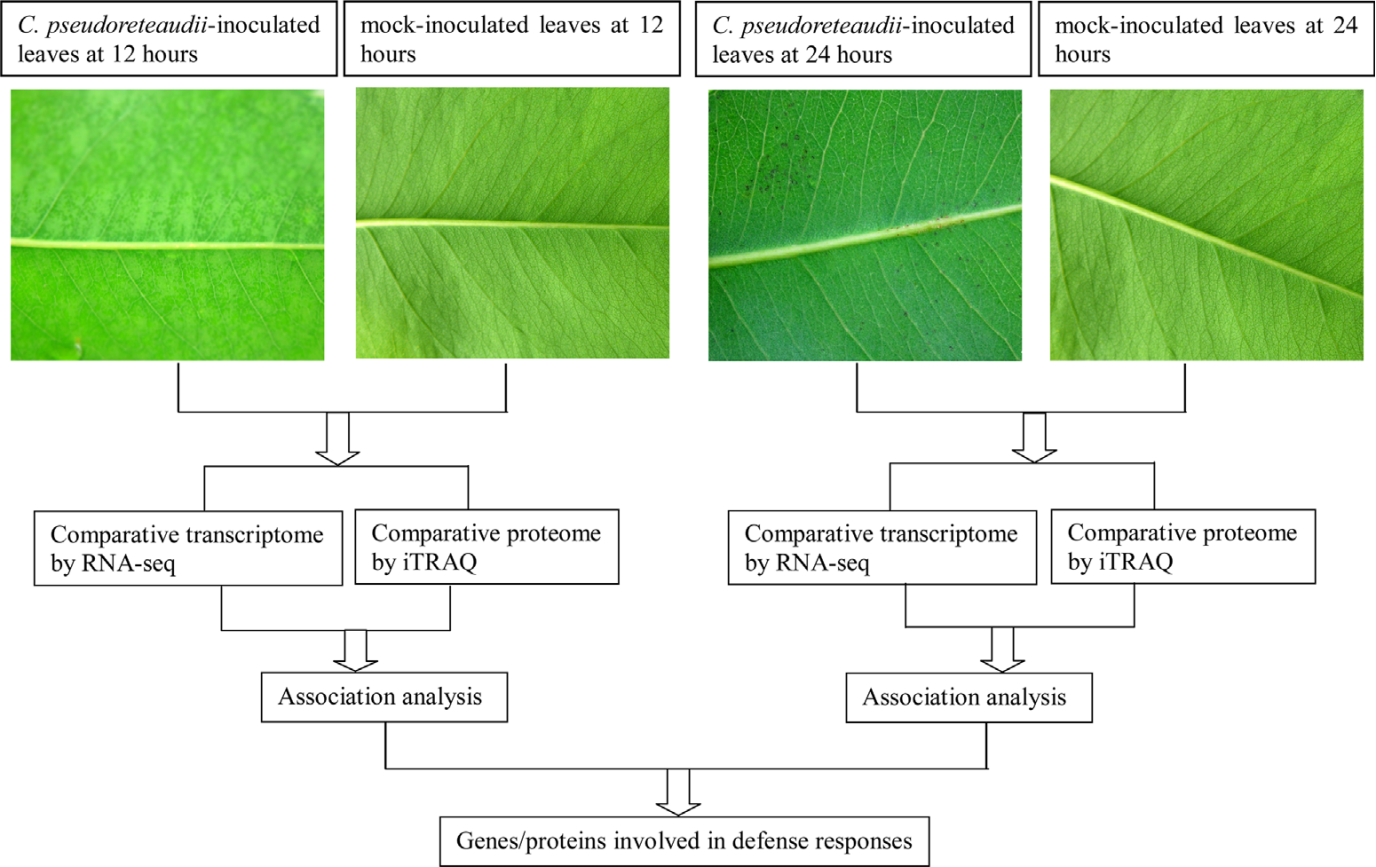
Comparative analysis of transcriptome and proteome between *C. pseudoreteaudii*-inoculated leaves and mock-inoculated leaves at 12 and 24 h.

The resistant clone *E.* *urophylla*×*E. tereticornis* M1 was employed as experimental material. The transcriptome and proteome of *Eucalyptus* leaves infected or not infected with *Calonectria pseudoreteaudii* at 12 and 24 h were investigated by RNA-seq and iTRAQ, respectively. Fig. 1 shows the experimental design used to get the data in this article and in [1].

### 1.2 RNA isolation, illumina sequencing and raw data processing

Total RNA was extracted from mixed leaves of five replications of each sample using RNAplant Plus Reagent DP437 (Tiangen, Beijing, China) according to the manufacturer׳s instructions. The concentration and quality of RNA were determined using an Agilent 2100 Bioanalyzer (Agilent Technologies, Santa Clara, CA, USA) (Supplementary Fig. 1 and Table 1, Sheet 1).

Total RNA extracts of “*E.* *urophylla*×*E. tereticornis M1*” were then sent to the Beijing Genomics Institute for RNA-Seq (BGI, Shenzhen, China). The construction of the cDNA library and sequencing were performed as previous report [2,3]. Briefly, mRNA was enriched by oligo(dT) beads (Dynabeads mRNA purification kit, Invitrogen), and then fragmented into short pieces (about 200 bp). The first strand cDNA was synthesized by random hexamer-primer, First Strand Master Mix and Super Script II reverse transcription (Invitrogen, USA). Buffer, dNTPs, RNase H and DNA polymerase I were added to synthesize the second strand. The double strand cDNA was purified with QiaQuick PCR Purification Kit (Qiagen, Germany) and subject to end-repair followed by addition of a single ‘A’ base and ligation of adapters. The acquired fragments was purified and enriched by PCR amplification (Reaction condition: 94 °C for 2 min; 94 °C for 15 s, 62 °C for 30 s, 72 °C for 30 s, 15 cycles;72 °C for 10 min). The generated library was validated by determining the average molecule length with Agilent 2100 Bioanalyzer and quantified by real-time quantitative PCR (Kapa biosystem, USA). The final library were amplified on cBot to generate the cluster on the flowcell by TruSeq SR Cluster Kit v3 (Illumina, USA), and sequenced pair end using TruSeq SBS kit on Illumina HiSeq 2000 system (Illumina, San Diego, CA, USA).

The resulting data were analyzed in a slightly modified version of the procedure as previous description [2]. FASTQ files containing 50 bp single-end RNA-Seq reads were mapped to the sequenced genome of *E. grandis* (http://phytozome.net/eucalyptus.php) using SOAP2 software allowing two base mismatches [4]. The gene expression level was calculated using the RPKM [5] method.

### 1.3 Identification and annotation of DEGs

To identify genes that were differentially expressed between pathogen-inoculated and mock-inoculated samples in the two stages, the False Discovery Rate (FDR) ≤0.001 and the absolute value |log2 Ratio|≥1 were set as the thresholds to judge the significance of differences in gene expression (Supplementary Table 2). Then, all DEGs were mapped to gene ontology terms in the database (GO, http://www.geneontology.org/) for functional annotation. Additionally, the DEGs were subjected to Kyoto Encyclopedia of Genes and Genomes database (KEGG, http://www.genome.jp/kegg/pathway.html) enrichment analysis to identify the main metabolic pathways and signal transduction pathways of DEGs using Blastall software.

### 1.4 Protein extraction and iTRAQ reagent labeling

The plant materials used for iTRAQ analysis were the same as those for RNA-Seq. Protein was extracted from each sample according to the method of Yang et al. [6]. The protein concentration and quality were determined using a Protein Assay Kit (Bio-Rad, Hercules, CA, USA) and confirmed with a 15% SDS-PAGE (Geneview, USA)(Supplementary Fig. 2 and Table 1, Sheet 2).

iTRAQ analysis was carried out as previous reports at Beijing Genomics Institute (BGI, Shenzhen, China) [6]. Briefly, after adjusting the pH to 8.5 with 1 M ammonium bicarbonate (Analytical grade reagents, China), total protein from each sample was reduced for 1 h at 56 °C by adding DL-Dithiothreitol (Amresco, USA) to 10 mM, and alkylated with 55 mM iodoacetamide (Sigma, USA) for 45 min at room temperature in the dark. Trypsin (Promega, USA) was then added to a final substrate/enzyme ratio of 20:1 (w/w). The digest was incubated at 37 °C for overnight. Every sample (100 μg) was then labeled using iTRAQ Reagent-8plex Multiplex Kit according to the manufacturer׳s instructions (Applied Biosystems, Foster City, CA, USA). Two pathogen-inoculated samples were labeled with iTRAQ tags 113 and 115, two control samples labeled with tags 117 and 119.

### 1.5 Strong cation-exchange fractionation

The labeled samples were mixed and lyophilized. They were then resuspended in 4 mL of solvent A (25% v/v acetonitrile, 25 mM NaH_2_PO_4_, pH 2.7)(Sigma, USA) and loaded into a Ultremex SCX column (4.6×250 mm) (Shimadzu LC-20AB HPLC). The peptide was eluted at 1 mL min^−1^ using solvent A for 10 min, 5–35% solvent B (25 mM NaH_2_PO_4_, 1 M KCl, 25% v/v acetonitrile, pH 2.7) for 11 min, and then 35–80% solvent B for 1 min. The eluted fractions were monitored through a UV detector at 214 nm. Fractions were collected every 1 min, and consecutive fractions with low peak intensity were combined. A total of twenty fractions were obtained, desalted using a Strata X C18 column (Phenomenex, USA) and then vacuum-dried.

### 1.6 Liquid chromatography–mass spectrometry (LC–MS/MS)

Each of the dried fractions was dissolved with solvent C (5% v/v acetonitrile, 0.1% Formic acid) (Sigma, USA) and centrifuged at 20,000 g for 10 min. The final concentration was 0.5 μg/μl. The peptide (8 μl) was loaded into a 2 cm C18 trap column (inner diameter 200 μm) on an Shimadzu LC-20 AD nano HPLC. The sample was loaded at 8 μl/min for 4 min, then eluted at 300 nl/min for 40 min with a gradient of 2–35% solvent D (95% v/v acetonitrile, 0.1% Formic acid), followed by a 5 min linear gradient to 80%, maintaining at 80% solvent D for 4 min, and then at solvent C for 1 min.

The eluted peptides were analyzed using nanoelectrospray ionization followed by tandem mass spectrometry (MS/MS) in an Q-Exactive (Thermo Fisher Scientific, San Jose, USA) coupled online to the HPLC. Intact peptides were detected in the Orbitrap with a resolution of 70,000. Peptides were selected for MS/MS using higher energy collision dissociation (HCD) operating mode with a normalized collision energy setting of 27%. A data-dependent procedure that alternated between one MS scan followed by fifteen MS/MS scans was applied for the three most abundant precursorions above a threshold ion count of 20,000 in the MS survey scan.

### 1.7 Data analysis

The MS spectra were analyzed by a thorough search using Mascot software (version 2.3.02, Matrix Science Inc, Boston, MA) against *E. grandis* database (JGI version 0.9, www.phytozome.org/Egrandis, 46,315 sequences). Search parameters were as followed: MS/MS ion search; trypsin enzyme; fragment mass tolerance 0.02 Da; monoisotopic mass values; variable modifications of Gln->pyro-Glu (N-term Q), oxidation (M) and iTRAQ8plex (Y); peptide mass tolerance 15 ppm; one max missed cleavage. To reduce false positive results, all data were reported based on a 95% confidence and false discovery rate (FDR) less than 1%. For quantitative analysis, a protein must have at minimum one unique peptide matches with iTRAQ ratios. A 1.2-fold cutoff value was used to identify up-regulated and down-regulated proteins with a *p*-value of less than 0.05. All the specific protein groups and their corresponding peptide list were presented in Supplementary Table 3. The information of protein quantitation was provided in Supplementary Table 4.

## Footnotes

Appendix A Supplementary data associated with this article can be found in the online version at doi:10.1016/j.dib.2014.12.008.
