# Supplementary figures and images for: Data for transcriptome and proteome analysis of Eucalyptus infected with Calonectria pseudoreteaudii
Source: Data Brief. 2015 Jan 9;3:24–8. doi: 10.1016/j.dib.2014.12.008 (PMC4509981; doi:10.1016/j.dib.2014.12.008)

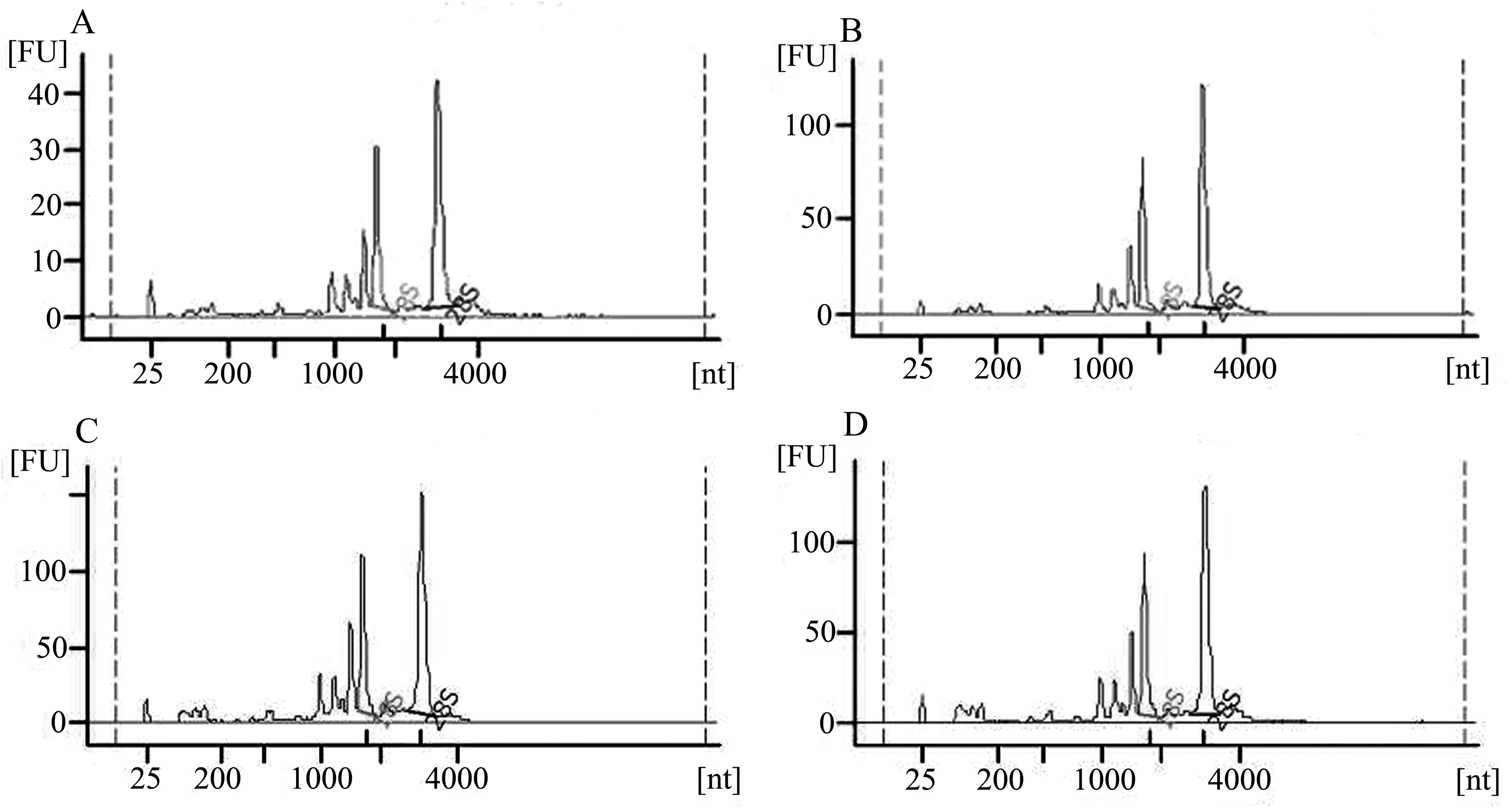

Supplement: Supplementary file 1 — Supplementary Material [file mmc1.zip › Supplementary Figure 1.tif]

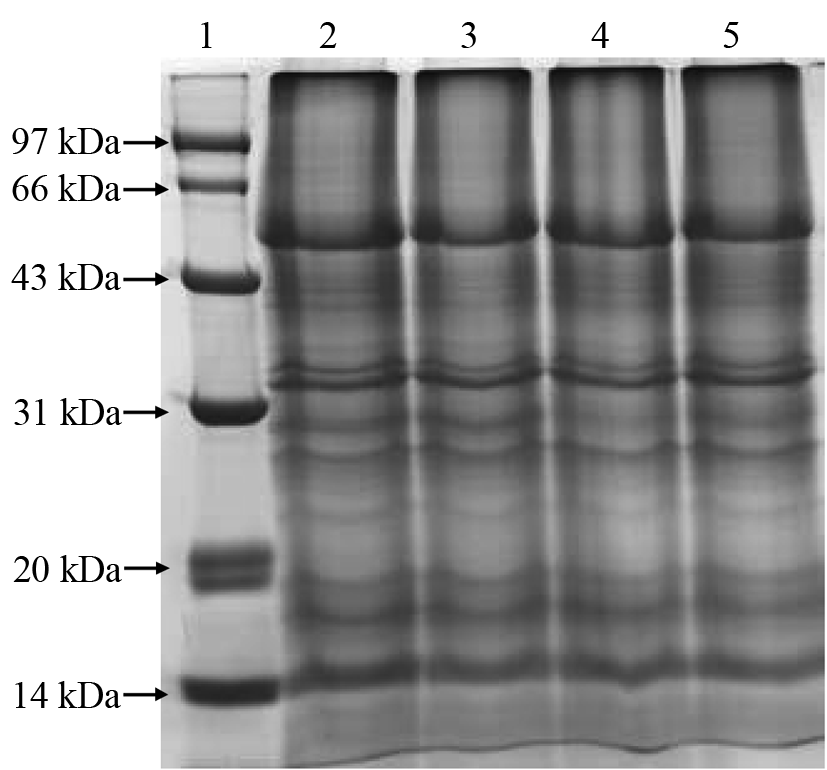

Supplement: Supplementary file 2 — Supplementary Material [file mmc2.zip › Supplementary Figure 2.tif]
